# Patient-Derived Organoids in Gastrointestinal Disease: Current Applications, Limitations, and Future Perspectives

**DOI:** 10.3390/ijms27156949

**Published:** 2026-08-02

**Authors:** Amanda Caruso, Yasmine Hamrouni, Antonella Delvecchio, Riccardo Memeo, Stefano Martinotti

**Affiliations:** 1 Department of Medicine and Surgery, LUM University “Giuseppe Degennaro”, Casamassima, 70010 Bari, Italy; 2 Clinical Pathology Unit, “F. Miulli” University Hospital, Acquaviva delle Fonti, 70021 Bari, Italy; 3 Unit of Hepato-Biliary and Pancreatic Surgery, Ecclesiastical Entity Regional General Hospital “F. Miulli” General Hospital, Acquaviva delle Fonti, 70010 Bari, Italy

**Keywords:** patient-derived organoids, gastrointestinal diseases, precision medicine, 3D cell culture, organoid-on-chip

## Abstract

The development of patient-derived organoids (PDOs) has substantially advanced the study of gastrointestinal diseases by providing three-dimensional human models that faithfully recapitulate the structural, molecular, and functional characteristics of native tissues. Unlike conventional two-dimensional cultures and animal models, intestinal organoids preserve epithelial architecture, cellular heterogeneity, and patient-specific genetic features, enabling more physiologically relevant investigations of gastrointestinal physiology and disease. Recent technological advances, including co-culture systems, organoid-derived monolayers, and organ-on-chip platforms, have further expanded their ability to model epithelial interactions with immune cells, stromal components, and the gut microbiota. These developments have facilitated mechanistic studies of epithelial barrier function, host–microbiota communication, microbial metabolites, and endocrine signaling, while also supporting translational applications in inflammatory bowel disease, infectious disorders, inherited gastrointestinal diseases, and gastrointestinal cancers. Moreover, patient-derived organoids have emerged as promising platforms for drug screening, biomarker discovery, precision medicine, and regenerative therapies. Despite these advances, several challenges remain, including limited representation of the native tissue microenvironment, lack of standardized culture protocols, scalability, and regulatory issues that currently restrict routine clinical implementation. This review summarizes recent progress in gastrointestinal organoid technology, highlighting current applications, emerging experimental platforms, and future perspectives for integrating organoid-based models into translational research and personalized medicine.

## 1. Introduction

The gastrointestinal tract represents a highly dynamic interface in which epithelial renewal, microbial exposure, immune signaling, and environmental stimuli continuously interact to maintain tissue homeostasis [1]. Disruption of these tightly regulated processes contributes to the development of a broad spectrum of gastrointestinal diseases, including inflammatory, infectious, metabolic, and neoplastic disorders. In particular, epithelial barrier dysfunction and altered host–microbiota interactions are increasingly recognized as central mechanisms driving chronic intestinal inflammation and disease progression [2]. Despite significant advances in molecular biology and translational gastroenterology, the study of gastrointestinal diseases remains limited by the lack of experimental systems capable of faithfully reproducing the structural and functional complexity of the human intestine [3]. Conventional two-dimensional (2D) cell cultures have historically provided accessible and reproducible platforms for investigating fundamental cellular processes; however, they fail to recapitulate the three-dimensional organization, cellular heterogeneity, and microenvironmental interactions that characterize native intestinal tissues [4,5,6]. Similarly, animal models only partially reflect human gastrointestinal physiology because of important interspecies differences and limited translational predictability [4]. In this context, the development of three-dimensional (3D) organoid cultures has represented a major advance in gastrointestinal research [5,6,7,8] (Supplementary Table S1). Organoids are self-organizing multicellular structures derived from adult stem cells or pluripotent stem cells that preserve key architectural, molecular, and functional features of the tissue of origin [9,10]. Beyond their structural resemblance to native tissues, intestinal organoids provide versatile experimental platforms to investigate dynamic epithelial responses to inflammation, microbial stimuli, metabolic alterations, and tumorigenesis [6]. Recent advances in organoid technology, including patient-derived organoids, co-culture systems, and organoid-on-chip platforms, have further expanded their translational relevance by enabling the study of host–microbiota interactions, immune crosstalk, and personalized therapeutic responses in a controlled human-relevant environment [11,12,13,14]. Importantly, emerging evidence suggests that intestinal organoids also represent valuable systems to investigate the gut–endocrine axis, a complex regulatory network linking epithelial sensing, microbial metabolites, and enteroendocrine signaling to systemic metabolic homeostasis [15]. Through the integration of epithelial, microbial, and endocrine pathways, organoid models are increasingly shifting gastrointestinal research from descriptive observations toward mechanistic and translational investigation. This review provides an overview of the translational applications of patient-derived intestinal organoids, with a focus on the integration of epithelial biology with immune, microbial, stromal, endocrine, and metabolic interactions. It discusses advanced co-culture systems, organoid-derived monolayers, and organ-on-chip platforms, highlighting their use in inflammatory, infectious, inherited, metabolic, and neoplastic gastrointestinal disorders. Mechanistic studies, functional assays, drug screening, biomarker discovery, regenerative strategies, and emerging clinical applications are considered to outline the current opportunities and remaining challenges of patient-derived organoid (PDO) technology [3,16].

## 2. Human Intestinal Organoids as Models of Gastrointestinal Diseases

The strength of intestinal organoids lies in their ability to reproduce not only epithelial architecture, but also the regulatory programs that sustain intestinal homeostasis and adaptation. Over the past decade, organoid technology has expanded rapidly across multiple areas of gastroenterology. A wide range of culture protocols has been developed, enabling the generation of organoids from both adult stem cells (ASCs) and pluripotent stem cells (PSCs), which recapitulate key structural and functional features of different regions of the gastrointestinal tract [17]. A major advance in the field was the demonstration that Lgr5^+^ intestinal crypt base columnar stem cells are capable of generating self-organizing epithelial structures in vitro, establishing the foundation for long-term intestinal organoid cultures [5]. These stem cell populations retain intrinsic developmental programs that regulate epithelial renewal and lineage specification through signaling pathways such as Wnt, Notch, and BMP. As a result, intestinal organoids preserve multiple differentiated epithelial cell types, including absorptive enterocytes, goblet cells, Paneth cells, and enteroendocrine cells, enabling the investigation of epithelial dynamics under both physiological and pathological conditions [18]. Importantly, patient-derived organoids retain not only tissue-specific genetic alterations, but also epigenetic signatures associated with inflammation and disease state, thereby preserving key features of the original tissue microenvironment [19]. Beyond their structural resemblance, intestinal organoids preserve fundamental epithelial functions, making them particularly suitable for investigating gastrointestinal physiology and disease [20]. The intestinal epithelium operates as a dynamic barrier that regulates nutrient absorption while protecting the host from luminal antigens and microorganisms. This barrier function depends on tightly coordinated interactions between epithelial cell types, junctional complexes, and the surrounding microbiota, while protecting the host from luminal antigens, toxins, and microorganisms [21]. This function depends on coordinated interactions between epithelial cell populations, junctional complexes, and microbial signals. Alterations in tight junction and adherens junction proteins, including occludin, claudins, ZO-1, and cadherins, are closely associated with barrier dysfunction and chronic inflammation in several gastrointestinal diseases [22]. In this context, organoid systems provide valuable platforms to investigate how epithelial integrity is disrupted under pathological conditions and how regenerative responses are activated following injury [23,24]. Recent technological advances have further expanded the biological relevance of PDOs. Co-culture systems integrating immune cells, stromal populations, microbial components, or neuronal elements increasingly allow the reconstruction of critical aspects of the intestinal microenvironment [25]. These approaches have demonstrated how bidirectional epithelial–immune–microbial communication regulates barrier integrity, inflammatory responses, epithelial regeneration, and metabolic adaptation [23]. Similarly, organoid-derived monolayers, air–liquid interface cultures, and organoid-on-chip systems provide direct access to the luminal compartment and enable more physiologically relevant modeling of microbial exposure and environmental stimuli [26,27]. These systems increasingly allow the reconstruction of key components of the GI microenvironment, including immune, stromal, neural, and microbial compartments.

## 3. PDO in Gastrointestinal Microenvironment

The intestinal epithelium is not merely a physical barrier but an active participant in immune surveillance and tissue homeostasis [28]. Under physiological conditions, continuous bidirectional communication between epithelial and immune cells coordinates tolerance toward commensal microorganisms while preserving the ability to mount effective responses against pathogens [29]. Disruption of this tightly regulated crosstalk contributes to the development of chronic inflammatory disorders, impaired tissue repair, and tumor progression [30]. A major limitation of conventional epithelial models has been the inability to faithfully reproduce the complex interactions occurring between the intestinal epithelium and the underlying immune compartment. While intestinal organoids accurately recapitulate epithelial architecture and cellular diversity, they initially lacked key components of the in vivo microenvironment, including hematopoietic, stromal, neural, and vascular cell populations. Recent advances in co-culture technologies have progressively addressed these limitations by enabling the integration of immune cells into organoid-based systems, thereby allowing a more comprehensive investigation of immune–epithelial communication [31,32]. To date, intestinal organoids have been successfully co-cultured with a wide range of immune populations, including T lymphocytes, macrophages, dendritic cells, neutrophils, innate lymphoid cells (ILCs), and intraepithelial lymphocytes [33]. These models have provided important insights into the mechanisms by which immune-derived signals regulate epithelial differentiation, barrier maintenance, and regenerative responses following injury [11]. Conversely, epithelial cells actively shape immune-cell behavior through the secretion of cytokines, chemokines, antimicrobial peptides, and other immunomodulatory mediators, establishing a dynamic feedback loop that is essential for intestinal homeostasis [34]. Among the immune populations investigated, innate lymphoid cells have emerged as critical regulators of epithelial function. In particular, type 3 innate lymphoid cells (ILC3s) contribute to epithelial regeneration through the production of IL-22, a cytokine that promotes stem-cell proliferation, enhances antimicrobial defense, and supports barrier integrity [34]. Co-culture systems combining intestinal organoids and ILCs have demonstrated that IL-22 signaling directly influences epithelial renewal and differentiation, highlighting its central role in mucosal repair processes [35]. Similarly, macrophage–organoid co-cultures have revealed how macrophage-derived cytokines modulate epithelial proliferation and inflammatory responses, whereas dendritic-cell models have provided insights into antigen presentation and immune activation within the intestinal mucosa [36]. The development of organoid-derived monolayers and air–liquid interface cultures has further expanded the study of immune–epithelial interactions [37]. These systems provide simultaneous access to both the apical and basolateral compartments, allowing immune cells to interact with epithelial surfaces in a manner that more closely resembles physiological conditions. Such approaches have proven particularly valuable for investigating host responses to enteric pathogens, cytokine-mediated epithelial injury, and mechanisms of immune-mediated barrier dysfunction [38]. Interestingly, immune–epithelial crosstalk does not occur in isolation but is strongly influenced by microbial-derived signals and metabolites [3]. Increasing evidence suggests that microbiota-dependent modulation of immune-cell activity can indirectly affect epithelial regeneration, barrier permeability, and secretory functions. Consequently, immune–organoid co-culture systems are becoming increasingly relevant for understanding how dysregulated interactions among epithelial, microbial, and immune compartments contribute to chronic inflammatory diseases and other gastrointestinal disorders. Beyond their relevance to inflammatory conditions, these models also offer significant translational opportunities. Patient-derived organoids combined with autologous immune cells are emerging as promising platforms for evaluating immune-targeted therapies, investigating mechanisms of treatment resistance, and identifying personalized therapeutic strategies. As organoid technologies continue to evolve, the integration of immune, stromal, and vascular components is expected to generate increasingly sophisticated models capable of reproducing the complexity of the intestinal microenvironment and its contribution to disease pathogenesis.

## 4. PDO in Host–Microbiota Interactions

PDO technology has transformed simplified epithelial cultures into increasingly sophisticated multicellular platforms capable of modeling the dynamic interactions underlying gastrointestinal pathophysiology [39,40]. In fact, host–microbiota interactions represent one of the expanding applications of technology. These features enable the investigation of host–microbe communication in a controlled and physiologically relevant human context. Recent methodological advances, including luminal microinjection, apical-out organoids, organoid-derived monolayers, air–liquid interface cultures, and microfluidic platforms such as HuMiX, have further expanded the ability to model direct microbial exposure while maintaining tissue-specific epithelial architecture [41,42]. These technological developments have enabled the establishment of a broad range of host–microbe co-culture systems involving commensal bacteria, pathogenic microorganisms, parasites, and enteric viruses. Infection models have provided valuable mechanistic insights into epithelial invasion, inflammatory signaling, barrier disruption, and tissue repair [17]. For example, exposure to pathogenic bacteria such as *Salmonella enterica* induces robust inflammatory responses characterized by activation of NF-κB signaling, disruption of tight-junction integrity, and increased epithelial permeability. In parallel, cytokine-mediated pathways play a critical role in epithelial defense, with IL-22 signaling promoting antimicrobial peptide production, epithelial regeneration, and barrier protection through activation of downstream regenerative programs [3]. In contrast, commensal microorganisms exert predominantly protective effects on epithelial homeostasis. Co-culture studies using PDO with *Lactobacillus reuteri* and *Lactobacillus plantarum* have demonstrated enhanced epithelial proliferation, increased stem-cell activity, and improved mucosal repair—effects that are partly mediated through IL-22 and STAT3 signaling pathways [43,44]. Similarly, exposure to *Lactobacillus rhamnosus* has been associated with enhanced Paneth-cell differentiation and increased epithelial regenerative capacity [45]. However, host–microbiota interactions remain highly context dependent. Even non-pathogenic strains of *Escherichia coli* can exert dual effects, promoting epithelial maturation and antimicrobial defense under physiological conditions while inducing oxidative stress and apoptosis in specific experimental settings [33]. Beyond acute infection, organoid systems have also provided important insights into the long-term consequences of microbial exposure. A landmark study demonstrated that repeated luminal exposure of human intestinal organoids to genotoxic pks+ *Escherichia coli* generated a characteristic mutational signature that was subsequently identified in colorectal cancer tissues. These findings provided direct evidence that chronic microbial exposure may contribute to carcinogenesis through sustained genotoxic stress and highlighted the potential of organoid systems to investigate microbiota-driven tumor initiation. Organoid-based models have also been successfully applied to the study of parasitic and viral infections. Comparative infection systems using *Giardia duodenalis* and *Toxoplasma gondii* revealed distinct pathogen-specific effects on epithelial barrier function, with *Giardia* causing marked disruption of tight-junction integrity and epithelial resistance, whereas *Toxoplasma* exerted more limited effects. Similarly, intestinal and gastric organoids have significantly advanced the understanding of SARS-CoV-2 pathogenesis. Studies using both bat-derived and human intestinal organoids demonstrated efficient viral infection and replication within the gastrointestinal epithelium, supporting the concept that the gastrointestinal tract may serve as an important route of viral transmission [33]. Notably, host–microbiota interactions occur within a complex multilayered barrier system that extends beyond the epithelial compartment alone [46]. Emerging organoid-based models increasingly incorporate components of the mucus barrier, immune compartment, stromal niche, and gut vascular barrier, enabling a more comprehensive investigation of how microbial signals influence tissue homeostasis, inflammatory activation, and disease progression. Advanced co-culture systems integrating immune and stromal cells have further demonstrated that microbial-derived signals regulate cytokine production, immune-cell maturation, epithelial regeneration, and tissue repair through bidirectional communication between epithelial, microbial, and immune compartments. Although direct host–microbe interactions play a central role in regulating epithelial homeostasis, many microbiota-associated effects are mediated indirectly through bioactive metabolites [3,47,48]. Although these experimental platforms have considerably expanded the study of host–microbiota interactions, each approach reproduces only selected aspects of the intestinal ecosystem. Luminal microinjection preserves the physiological apical orientation and three-dimensional epithelial architecture, but it is technically demanding, difficult to scale, and generally unsuitable for prolonged or repeated microbial exposure. Apical-out organoids provide direct access to the luminal surface and facilitate microbial inoculation, although polarity reversal may modify epithelial organization and differentiation. Organoid-derived monolayers and air–liquid interface cultures enable quantitative assessment of barrier permeability and transepithelial transport, but partially lose the spatial organization and cellular gradients of three-dimensional cultures. In contrast, microfluidic platforms, including HuMiX and gut-on-chip systems, support continuous perfusion, oxygen gradients, mechanical stimulation, and more prolonged microbial colonization. However, their technical complexity, cost, limited throughput, and lack of standardized protocols currently restrict their widespread implementation. Therefore, the selection of the experimental platform should be guided by the biological question, as no single model currently reproduces microbial colonization, barrier physiology, immune interactions, chronic exposure, and anaerobic conditions simultaneously.

### Microbiota-Derived Metabolites and Epithelial Signaling

The biological effects of the gut microbiota are not solely mediated by direct interactions between microorganisms and host tissues but also by a diverse repertoire of bioactive metabolites generated through microbial metabolism [49]. Increasing evidence indicates that these molecules function as key signaling mediators capable of regulating epithelial differentiation, stem-cell activity, barrier integrity, immune responses, and tissue regeneration. Consequently, microbial metabolites are increasingly recognized as critical intermediaries linking luminal microbial composition to host physiology [49,50]. Intestinal organoids have emerged as valuable platforms for investigating the epithelial responses to microbial-derived metabolites under controlled experimental conditions. Unlike in vivo models, organoids allow the direct assessment of specific metabolites on defined epithelial populations while minimizing confounding systemic influences [51]. This approach has facilitated the identification of signaling pathways through which microbial metabolites regulate intestinal homeostasis and disease progression. Among the most extensively studied microbial products are short-chain fatty acids (SCFAs), including acetate, propionate, and butyrate. Generated through bacterial fermentation of dietary fibers, SCFAs contribute to epithelial energy metabolism and influence multiple cellular processes, including proliferation, differentiation, and barrier maintenance [46,52]. Organoid-based studies have demonstrated that SCFAs can modulate stem-cell activity and epithelial renewal while simultaneously regulating inflammatory responses through epigenetic and transcriptional mechanisms [53,54,55]. These observations support the concept that microbial metabolism directly contributes to the maintenance of intestinal homeostasis [22]. Beyond microbiota-derived metabolites, intestinal organoids have also been used to investigate the effects of nutrient-induced metabolic stress. Chronic exposure of mouse small intestinal organoids to palmitate has been shown to alter epithelial differentiation, including changes in the expression of enterocyte- and goblet cell-associated markers such as Hes1 and Muc2. Palmitate exposure also impaired the enteroendocrine lineage by reducing the expression of the enteroendocrine progenitor marker Ngn3 and the I-cell marker cholecystokinin, resulting in fewer CCK-positive cells and reduced CCK secretion. These findings illustrate how intestinal organoids can be used to dissect nutrient-induced lipotoxicity and its effects on epithelial and enteroendocrine homeostasis, extending organoid applications to metabolic disorders associated with obesity and diabetes [56]. Beyond SCFAs, several additional microbial metabolites have been shown to exert specific effects on epithelial cell fate and regenerative programs. For example, succinate promotes differentiation of tuft cells, a specialized epithelial population involved in chemosensing and type 2 immune responses [57]. Similarly, secondary bile acids have been shown to influence intestinal stem-cell behavior and epithelial regeneration while modulating the function of Paneth cells, which are essential for maintaining the stem-cell niche. Other bacterial metabolites can regulate endoplasmic reticulum stress responses, antimicrobial defense pathways, and epithelial differentiation programs, further highlighting the complexity of metabolite-mediated host–microbe communication. Recent studies have also revealed that microbial metabolites influence epithelial signaling networks that extend beyond local tissue homeostasis. Through interactions with pathways such as Wnt/β-catenin, Notch, mTOR, and aryl hydrocarbon receptor (AhR) signaling, microbiota-derived molecules can regulate cellular plasticity, regenerative responses, and adaptation to environmental stress [58]. These mechanisms are increasingly implicated in the pathogenesis of inflammatory bowel disease, colorectal cancer, and metabolic disorders, emphasizing the importance of understanding how microbial metabolic activity shapes epithelial behavior [29]. Furthermore, emerging evidence indicates that microbial metabolites are also capable of regulating enteroendocrine cell differentiation and function. Short-chain fatty acids, bile acids, and tryptophan-derived metabolites can stimulate hormone secretion and modulate signaling pathways involved in glucose homeostasis, appetite regulation, and energy expenditure [59,60]. As a result, the intestinal epithelium as a sensory interface capable of translating microbial-derived signals into endocrine outputs with systemic consequences [58]. Taken together, these findings position microbial metabolites as central mediators of host–microbiota communication [57]. By enabling mechanistic investigation of metabolite-specific effects on epithelial and enteroendocrine cells, intestinal organoids provide a powerful experimental framework for dissecting the molecular pathways that connect microbial ecology, intestinal physiology, and systemic metabolic regulation. Despite their value for dissecting metabolite-specific epithelial responses, conventional organoids primarily model direct local effects and do not reproduce metabolite absorption, systemic distribution, hepatic transformation, or feedback signals from distant metabolic organs. Moreover, differences in organoid differentiation status, culture medium, metabolite concentration, exposure duration, and passage number may contribute to apparently divergent findings across studies. Standardized exposure protocols and the integration of organoids with microfluidic and multi-organ platforms will therefore be required to distinguish physiologically relevant signaling from culture-specific effects.

## 5. Advanced Co-Culture and Organ-on-Chip Systems

**Figure 1:**
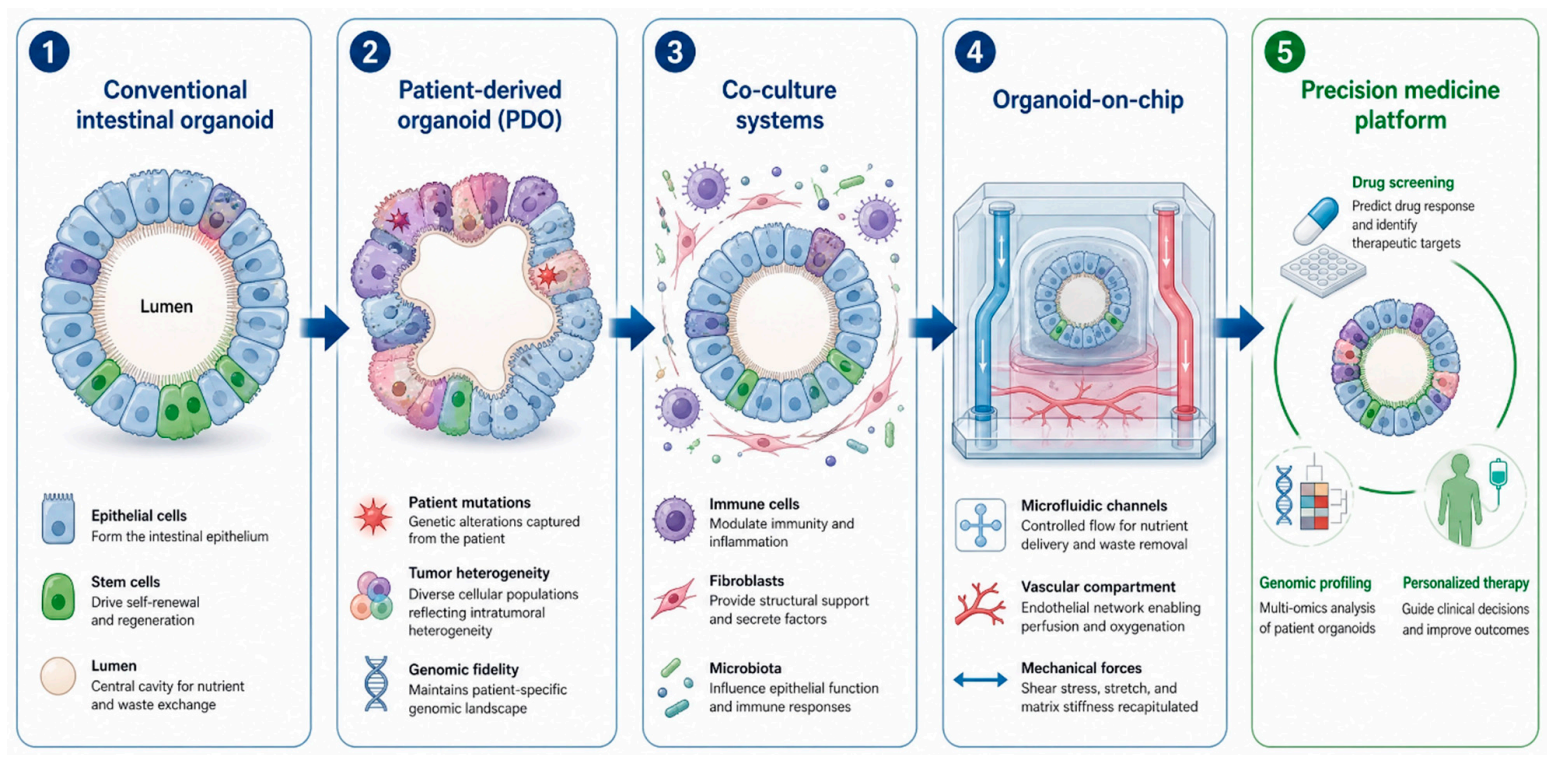
Evolution of intestinal organoid platforms. Schematic overview of the progressive development of intestinal organoid technology, from conventional epithelial organoids to patient-derived organoids (PDOs), multicellular co-culture systems, organ-on-chip platforms, and precision medicine applications, including disease modeling, drug screening, biomarker discovery, and regenerative medicine.

While conventional intestinal organoids have substantially improved the study of epithelial biology, they remain simplified representations of the intestinal mucosa. In their original configuration, organoids lack many of the cellular and physical components that shape tissue function in vivo, including immune cells, fibroblasts, neurons, endothelial cells, vascular networks, and mechanical stimuli. As a result, recent efforts have increasingly focused on developing multicellular organoid systems capable of reproducing the complexity of the intestinal microenvironment [3]. One of the major advances in the field has been the incorporation of stromal and mesenchymal cell populations into organoid cultures. Fibroblasts and subepithelial mesenchymal cells provide essential niche factors, including Wnt ligands, R-spondins, and BMP modulators, which regulate stem-cell maintenance, epithelial differentiation, and tissue regeneration [46]. Co-culture studies have demonstrated that mesenchymal populations actively influence epithelial growth and function, highlighting the importance of epithelial–stromal communication in maintaining intestinal homeostasis [57]. Increasing attention has also been directed toward the integration of neural components. The enteric nervous system (ENS) exerts profound effects on epithelial turnover, intestinal motility, immune responses, and secretion, and is increasingly recognized as an important contributor to gastrointestinal disease [61]. Recent studies have successfully generated innervated intestinal organoids through the incorporation of neural crest-derived cells, resulting in the formation of functional neuroglial networks that resemble components of the myenteric and submucosal plexuses [62]. These models provide unprecedented opportunities to investigate epithelial–neural communication and mechanisms underlying the gut–brain axis [57]. Similarly, the development of vascularized organoids represents an important step toward reproducing the physiological organization of intestinal tissues [63,64]. By incorporating endothelial and mesenchymal populations, researchers have generated organoid systems capable of mimicking aspects of the gut vascular barrier and tissue perfusion [65]. Such models are particularly relevant for studying nutrient transport, immune-cell trafficking, microbial translocation, and systemic inflammatory responses. Furthermore, vascularized organoids may improve the translational potential of regenerative medicine approaches by enhancing tissue maturation and engraftment following transplantation [61]. Beyond the integration of specific cellular compartments, microengineering technologies have further transformed the organoid field [57]. Organ-on-chip platforms combine organoid cultures with microfluidic systems that reproduce key physiological parameters, including fluid flow, oxygen gradients, nutrient exchange, and mechanical stimulation [61]. Unlike conventional static cultures, these devices enable continuous environmental control and permit the long-term co-culture of epithelial cells with immune populations, stromal cells, and anaerobic microorganisms under conditions that more closely resemble the in vivo intestinal environment [57] (Figure 1). Particularly noteworthy are gut-on-chip models, which have emerged as powerful tools for studying host–microbiota interactions, epithelial barrier function, inflammatory responses, and drug absorption [66,67,68,69]. By integrating multiple cellular compartments within a dynamic microfluidic environment, these systems overcome several limitations of traditional organoid cultures and provide enhanced experimental reproducibility [70,71,72,73]. Their ability to model tissue-level interactions in real time makes them especially attractive for translational applications, including drug screening, toxicity testing, and personalized medicine [67]. For example, gut-on-chip models have demonstrated superior capability compared with static PDOs in reproducing anaerobic microbial colonization, physiological fluid flow, cyclic mechanical strain, and long-term host–microbiota interactions in IBD and enteric infection models. Overall, these advances are progressively transforming intestinal organoids from epithelial-only cultures into increasingly sophisticated multicellular platforms capable of reproducing key aspects of the intestinal microenvironment [72]. The integration of immune, stromal, neural, vascular, and microbial components is expected to further improve the physiological relevance of organoid models and expand their applications in disease modeling, regenerative medicine, and precision therapeutics [57]. Compared with conventional static PDO cultures, gut-on-chip systems provide several functional advantages in disease modeling. In IBD, continuous perfusion and mechanical stimulation better reproduce epithelial barrier dynamics and inflammatory responses under physiological flow conditions. Likewise, in infectious disease models, microfluidic platforms support prolonged host–microbiota co-culture by maintaining oxygen gradients and, in selected systems, anaerobic conditions that are difficult to achieve using static organoids. These characteristics enable more physiologically relevant investigation of microbial colonization, barrier dysfunction, and host immune responses. Beyond reproducing local intestinal physiology, emerging multi-organ microphysiological systems are beginning to address one of the major limitations of conventional intestinal PDOs, namely the inability to model systemic inter-organ communication. In particular, gut–liver-on-chip platforms offer the opportunity to investigate bidirectional signaling mediated by microbial metabolites, bile acids, inflammatory mediators, and xenobiotics, thereby providing a more physiologically relevant framework for studying the gut–liver axis in metabolic diseases and drug metabolism. Furthermore, recent advances in the development of vascularized liver organoids and liver organoid-on-chip systems are expected to facilitate the integration of physiologically relevant hepatic counterparts into future multi-organ platforms, further improving the modeling of systemic host–microbiota interactions [74]. The progressive incorporation of additional cellular and physical components improves physiological relevance but also introduces new sources of variability. Multicellular co-cultures and organ-on-chip systems require precise control of cell ratios, differentiation states, media compatibility, fluid dynamics, and oxygen conditions. Consequently, increasing model complexity may reduce reproducibility, throughput, and inter-laboratory comparability. The most sophisticated platform is therefore not necessarily the most appropriate for every application, and model selection should balance biological complexity with robustness, scalability, and compatibility with the intended functional readout.

## 6. PDOs in Gastrointestinal Diseases

### 6.1. Inflammatory Bowel Disease

Inflammatory bowel diseases (IBD), including Crohn’s disease (CD) and ulcerative colitis (UC), are chronic relapsing disorders characterized by a complex interplay among genetic susceptibility, epithelial barrier dysfunction, immune dysregulation, environmental factors, and alterations in the gut microbiota [75]. Although considerable progress has been made in understanding IBD pathogenesis, the relative contribution of epithelial-intrinsic defects remains incompletely understood. Intestinal organoids have emerged as powerful tools for addressing this challenge, as they preserve many of the molecular, genetic, and functional characteristics of the tissue of origin while allowing the study of epithelial biology independently of systemic immune influences. Patient-derived organoids established from individuals with UC and CD retain disease-associated transcriptional and epigenetic signatures even after prolonged ex vivo culture. These models have revealed persistent alterations in epithelial differentiation, barrier integrity, antimicrobial defense mechanisms, and regenerative responses, supporting the concept that epithelial dysfunction is not merely a consequence of inflammation but may actively contribute to disease development and progression [76,77]. In particular, organoid studies have demonstrated abnormalities in tight-junction organization, mucus production, and epithelial repair pathways that closely resemble alterations observed in vivo. Organoid systems have also provided important insights into the effects of inflammatory cytokines on epithelial homeostasis. Exposure of intestinal organoids to TNF-α, IFN-γ, IL-17, and IL-22 has revealed profound effects on stem-cell function, epithelial proliferation, differentiation, and barrier maintenance. While transient activation of these pathways contributes to tissue repair and host defense, persistent cytokine signaling may impair regenerative programs and promote chronic epithelial injury. In this regard, organoid-based co-culture systems integrating immune cells have significantly advanced the understanding of immune–epithelial crosstalk during intestinal inflammation [35]. Beyond mechanistic investigations, organoids are increasingly being used to evaluate therapeutic responses in a patient-specific manner [46,75]. Several studies have demonstrated that patient-derived organoids can predict responses to biological therapies and small-molecule treatments, supporting their potential application in precision medicine [75]. By combining functional assays with genomic and transcriptomic analyses, organoid platforms may help identify biomarkers of treatment response and facilitate individualized therapeutic strategies. More recently, attention has shifted toward the use of intestinal organoids as regenerative therapies for IBD [78]. Since defective mucosal healing represents a major determinant of disease progression and relapse, transplantation of epithelial stem-cell-derived organoids has emerged as a promising strategy to restore epithelial integrity and promote tissue repair [76]. Experimental studies have demonstrated successful engraftment of transplanted organoids into damaged intestinal mucosa, resulting in epithelial reconstruction and functional recovery [78]. Building on these findings, autologous organoid-based approaches are currently being explored as potential regenerative therapies capable of enhancing mucosal healing while minimizing the risk of immune rejection [79,80,81]. Despite these encouraging results, several biosafety concerns must be addressed before clinical translation. Potential risks include incomplete epithelial differentiation, persistence of proliferative stem or progenitor cells, culture-acquired genomic alterations, and inappropriate engraftment, all of which could compromise long-term safety following transplantation. Therefore, rigorous preclinical validation, genomic stability assessment, and long-term post-transplantation monitoring will be essential before organoid-based regenerative therapies can be routinely applied in patients. Altogether, these studies highlight the value of intestinal organoids as versatile platforms for investigating IBD pathogenesis, evaluating therapeutic responses, and developing regenerative strategies. By preserving patient-specific epithelial characteristics while allowing precise experimental manipulation, organoid systems are increasingly bridging the gap between mechanistic research and personalized clinical application. Nevertheless, most organoid-based IBD studies remain preclinical, and differences in biopsy location, inflammatory status, culture conditions, passage number, and experimental readouts complicate direct comparison among studies. Although PDOs can preserve selected epithelial-intrinsic disease features, the extent to which ex vivo phenotypes predict treatment response or mucosal healing in patients still requires prospective validation. Standardized functional endpoints and multicenter studies will therefore be essential before PDO-based assays can be incorporated into routine IBD clinical workflows.

### 6.2. Infectious Gastrointestinal Diseases

Unlike conventional immortalized cell lines, PDO reproduce key features of the intestinal mucosa, enabling the investigation of pathogen entry, replication, epithelial injury, and host defense mechanisms within a controlled experimental environment. One of the earliest and most impactful applications of organoid technology in infectious disease research was the establishment of models for pathogens that were previously difficult or impossible to culture in vitro [82]. PDO have successfully supported infection by a wide range of enteric viruses, including human noroviruses, rotaviruses, enteroviruses, and SARS-CoV-2 [83,84,85,86]. These systems have provided important insights into viral tropism, replication dynamics, and epithelial responses to infection, while also facilitating the evaluation of antiviral compounds and therapeutic interventions [87]. In particular, infection-associated activation of NF-κB signaling, cytokine production, and tight-junction remodeling have been extensively characterized using organoid-based systems.

### 6.3. Genetic and Rare Gastrointestinal Disorders

Beyond inflammatory and infectious diseases, intestinal organoids have emerged as powerful platforms for investigating rare and genetically determined gastrointestinal disorders. One of the interesting applications of intestinal PDO has been in cystic fibrosis (CF) [88]. Mutations in the cystic fibrosis transmembrane conductance regulator (CFTR) gene impair epithelial ion transport, leading to abnormal fluid secretion and mucosal dysfunction in multiple organs, including the gastrointestinal tract [89]. Intestinal PDO derived from patients with CF faithfully reproduce defective CFTR activity and can be functionally assessed using the forskolin-induced swelling (FIS) assay [90,91,92]. In this system, activation of CFTR induces fluid secretion into the organoid lumen, resulting in organoid expansion, whereas CF organoids exhibit markedly reduced swelling responses. Importantly, FIS assays have demonstrated strong correlations with clinical outcomes and have become valuable tools for predicting individual responses to CFTR-modulating therapies, representing one of the earliest successful examples of organoid-guided precision medicine [88,89,93]. Despite its clinical utility, the FIS assay also presents technical limitations. Quantification of organoid swelling may be influenced by variability in organoid size, morphology, image acquisition, and analytical methods, while automated high-throughput implementation remains technically challenging. Continued efforts toward assay standardization and automated image analysis will be important to further improve reproducibility and facilitate broader clinical adoption. Organoid models have also contributed to the study of celiac disease, a chronic immune-mediated enteropathy triggered by dietary gluten in genetically susceptible individuals [94,95,96]. Although the pathogenesis of celiac disease involves complex interactions among epithelial cells, immune populations, and environmental factors, patient-derived organoids have provided important insights into epithelial-intrinsic responses to inflammatory stimuli and barrier dysfunction [97]. These systems have revealed alterations in epithelial differentiation, regenerative capacity, and stress-response pathways, supporting the concept that epithelial abnormalities contribute to disease susceptibility and progression [98]. More recently, organoid technology has been applied to a growing number of monogenic intestinal disorders, including congenital diarrheal diseases, epithelial transport defects, and disorders affecting intestinal development [99,100,101]. Genome-editing approaches combined with patient-derived organoids have enabled the functional characterization of disease-causing mutations and facilitated the identification of pathogenic mechanisms that are difficult to investigate using conventional models [102]. In parallel, gene-correction strategies based on CRISPR/Cas9 have demonstrated the feasibility of restoring normal epithelial function in selected genetic disorders, highlighting the potential of organoids as platforms for future regenerative and gene-based therapies [103]. The ability to integrate patient-specific genetics with functional readouts represents a major advantage of organoid technology in rare disease research. Collectively, these applications illustrate how intestinal organoids have transformed the study of rare gastrointestinal diseases from descriptive genetic analyses to functional and translational investigation. Their capacity to model patient-specific phenotypes and predict therapeutic responses positions organoid technology as a promising platform for the development of individualized treatment strategies in inherited gastrointestinal disorders [103].

### 6.4. PDO in Gastrointestinal Cancers

Organoids derived from gastrointestinal tumors, commonly referred to as PDOs, have become an increasingly valuable tool in cancer research. By retaining key features of the original tumor, including its genetic profile, cellular composition, and, to a certain extent, its histological organization, these models provide a more faithful representation of human disease compared to conventional cell lines [2,9]. As a result, PDOs are now widely used to investigate cancer biology and to test therapeutic strategies, including chemotherapy and targeted treatments. Their application spans multiple gastrointestinal malignancies, where they are increasingly contributing to a more personalized approach to cancer management [2,11,104].

#### 6.4.1. Colorectal Cancer

Colorectal cancer (CRC) is the most extensively studied model in organoid research and, in many ways, represents the setting in which this technology has reached its highest level of maturity. The first long-term organoid cultures were successfully established from intestinal epithelium, providing a foundation for the development of patient-derived tumor models [105]. Based on this, CRC-derived organoids have been shown to closely resemble the tumors from which they originate. As demonstrated in the establishment of living biobanks, these models retain not only the histological features of the primary lesion but also its genomic landscape, such as common alterations in genes *APC*, *KRAS*, and *TP53*, together with the broader mutational heterogeneity that characterizes colorectal cancer. Another relevant aspect is that CRC organoids maintain intra-tumoral heterogeneity over time. As highlighted by transcriptomic analyses, variability can be observed both between different patients and within individual organoid cultures, reflecting the complex clonal architecture of the original tumor. This makes them particularly useful for studying tumor evolution and mechanisms of resistance. From a functional perspective, CRC organoids have proven to be highly adaptable experimental systems. They can be expanded, cryopreserved, and used for large-scale drug screening approaches. In fact, these models allow the identification of genotype–drug associations and differential sensitivities across patients. Importantly, the translational relevance of CRC organoids has been supported by studies comparing ex vivo drug responses with clinical outcomes. Patient-derived organoids have been shown to recapitulate treatment responses observed in patients, including both sensitivity and resistance to chemotherapy and targeted agents, highlighting their potential role as predictive tools in precision oncology. Consistently, recent analyses of the field indicate that colorectal organoids are increasingly being used not only for disease modeling but also for drug screening and personalized medicine applications, reflecting a broader shift toward functional precision oncology [106].

#### 6.4.2. Gastric Cancer

Gastric organoids have emerged as a valuable model to study gastric carcinogenesis, as they allow the investigation of both epithelial transformation and host–pathogen interactions, particularly those involving *Helicobacter pylori* [107]. Unlike traditional in vitro systems, these models retain key features of the gastric epithelium and enable the analysis of early events in tumor initiation within a controlled environment.

In this context, gastric organoids have been used to explore the molecular mechanisms underlying malignant transformation, including alterations in signaling pathways such as Wnt/β-catenin and TP53. These pathways play a central role in regulating epithelial proliferation, differentiation, and genomic stability, and their dysregulation represents a critical step in gastric tumorigenesis. In addition, organoid systems make it possible to investigate how chronic inflammation, often driven by persistent *H. pylori* infection, contributes to cancer development by inducing epithelial damage, genomic instability, and aberrant signaling activation [107]. Importantly, gastric organoids also provide a platform to study the direct interaction between pathogens and epithelial cells. Infection models have shown that *H. pylori* can alter epithelial cell polarity, disrupt tight junctions, and modulate gene expression programs associated with tumor progression, offering insights into the link between infection and carcinogenesis [108]. Beyond mechanistic studies, gastric cancer organoids are increasingly used for translational applications. They preserve tumor heterogeneity and patient-specific molecular features, making them suitable for drug screening and for evaluating responses to targeted therapies. This approach may help identify predictive biomarkers and support the development of more personalized treatment strategies [12,109]. Overall, gastric organoids represent a powerful and flexible system to investigate both the early stages of tumor development and clinically relevant aspects of gastric cancer biology.

#### 6.4.3. Pancreatic Cancer

Pancreatic ductal adenocarcinoma (PDAC) organoids have attracted significant attention, reflecting the aggressive biology and poor prognosis of this disease. In recent years, PDOs have emerged as a robust model system, as they retain key molecular and phenotypic features of the original tumor, including common driver mutations such as *KRAS*, *TP53*, *CDKN2A*, and *SMAD4*, which are central to PDAC pathogenesis [110].

Unlike conventional models, pancreatic organoids enable the study of tumor development across different stages of disease progression. By introducing specific genetic alterations using gene-editing technologies such as CRISPR-Cas9, normal pancreatic organoids can be progressively transformed into neoplastic models, allowing the reconstruction of stepwise tumorigenesis and the investigation of early oncogenic events [111]. This approach has provided important insights into how combinations of driver mutations cooperate to promote malignant transformation. In addition to genetic modeling, pancreatic organoids offer a valuable platform to investigate the tumor microenvironment. Although classical organoid systems are primarily epithelial, co-culture strategies with cancer-associated fibroblasts and immune cells have been developed to better recapitulate stromal interactions, which play a critical role in PDAC progression, desmoplasia, and therapeutic resistance. From a translational perspective, PDAC organoids have been widely used for drug screening and functional precision medicine approaches. These models preserve inter-patient heterogeneity and have been shown to recapitulate individual responses to chemotherapy, including sensitivity and resistance to standard regimens such as FOLFIRINOX and gemcitabine-based therapies [110]. Importantly, organoid-based drug testing has demonstrated promising correlations with clinical outcomes, supporting their potential role in guiding patient-specific treatment strategies. This is particularly relevant in PDAC, which is characterized by a dense stromal environment and marked therapeutic resistance.

Furthermore, pancreatic organoids provide an opportunity to investigate mechanisms of treatment resistance, including drug-induced selection of resistant clones and adaptive cellular responses. This is particularly relevant in PDAC, where intrinsic and acquired resistance remains a major clinical challenge. By enabling longitudinal studies and repeated drug exposure, organoids allow the identification of therapeutic vulnerabilities and potential combination strategies to overcome resistance. Overall, pancreatic organoids represent a powerful platform that integrates genetic modeling, microenvironmental interactions, and translational applications, making them particularly valuable for advancing both the biological understanding and clinical management of PDAC. Together, these findings highlight how organoid models are shifting gastrointestinal research from descriptive observations toward functional and mechanistic understanding, with increasing relevance for clinical translation [112].

#### 6.4.4. Other Gastrointestinal Malignancies

Organoid models have also been successfully established for a broader range of gastrointestinal malignancies, including esophageal, hepatobiliary, and small intestinal cancers. Although these models are generally less developed than those for colorectal cancer, they have significantly expanded the applicability of organoid technology across the digestive tract [113].

These systems provide the opportunity to investigate tumor biology in a tissue-specific context, allowing comparative analyses of molecular pathways, differentiation states, and therapeutic responses across different anatomical sites. In particular, organoids derived from esophageal and gastric junction cancers have been used to study epithelial plasticity and early neoplastic transformation, while hepatobiliary organoids have contributed to understanding the heterogeneity of liver and biliary tract malignancies [109,114].

Importantly, organoid models are especially valuable in the study of rare gastrointestinal tumors, where access to representative experimental systems is often limited. By preserving patient-specific genetic and phenotypic features, these models enable the investigation of disease mechanisms that are otherwise difficult to capture using conventional approaches [113].

From a translational perspective, emerging evidence suggests that organoids derived from these less common malignancies can also be used for drug screening and the identification of novel therapeutic targets. Although still in earlier stages of development, these platforms hold considerable promise for extending precision medicine approaches beyond the most common gastrointestinal cancers [12,115].

Overall, the development of organoids across a wide spectrum of gastrointestinal malignancies highlights their versatility and supports their growing role in both basic and translational cancer research.

### 6.5. Translational Applications of Patient-Derived Organoids in Precision Oncology

Beyond their role as experimental models, patient-derived organoids (PDOs) are emerging as promising tools to support precision oncology. By preserving the histopathological architecture, genomic landscape, and intratumoral heterogeneity of the original tumor, PDOs serve as patient-specific avatars that enable ex vivo assessment of drug sensitivity while closely recapitulating individual tumor biology [12]. Unlike molecular profiling alone, functional drug testing using PDOs provides direct evidence of tumor response or resistance to therapeutic agents, thereby capturing the biological complexity underlying treatment efficacy. Growing evidence supports the clinical relevance of this approach. In a landmark study, Vlachogiannis et al. demonstrated a strong correlation between PDO drug responses and clinical outcomes in patients with metastatic gastrointestinal cancers, highlighting the potential of organoid-based functional testing to predict therapeutic efficacy [6]. Similarly, Ooft et al. demonstrated the feasibility of using PDO-based drug sensitivity testing to predict chemotherapy response in patients with metastatic colorectal cancer, supporting its potential as a complementary tool for individualized treatment selection [116]. Together, these studies suggest that combining functional drug screening with genomic profiling may improve patient stratification, particularly in gastrointestinal malignancies characterized by marked molecular heterogeneity. Beyond gastrointestinal oncology, patient-derived organoids are increasingly being applied to investigate treatment resistance and to validate targeted therapeutic strategies in other malignancies, highlighting the broad translational potential of these models [117]. Collectively, these findings support the integration of PDO-based functional assays into precision oncology workflows, where they may complement molecular profiling and contribute to more individualized therapeutic decision-making. As prospective clinical evidence continues to accumulate, PDOs are expected to play an increasingly important role in bridging preclinical research and clinical practice. A summary of the current applications, strengths, and major limitations of PDOs in gastrointestinal diseases is provided in Table 1. Despite encouraging correlations between ex vivo PDO responses and patient outcomes, organoid-based predictions are not universally concordant with clinical responses. Differences may arise from the absence of systemic pharmacokinetics, vascular delivery, stromal desmoplasia, immune-mediated effects, and treatment-induced changes occurring in vivo. In addition, variability in tissue sampling, culture establishment, passage number, drug exposure schedules, and response thresholds limits comparisons among studies. Prospective multicenter validation and standardized drug-testing pipelines are therefore required before PDO assays can be routinely used to guide therapeutic decisions.

## 7. Current Limitations and Future Perspectives

Despite their remarkable progress and rapidly expanding translational applications, intestinal organoids still present important biological, technical, and regulatory limitations that currently restrict their widespread implementation in both research and clinical practice [116]. Addressing these challenges will be essential for establishing organoids as standardized and reliable platforms for precision medicine (Table 1). One of the major limitations concerns the lack of standardized culture methodologies. Differences in tissue processing, culture media composition, growth-factor supplementation, and passaging protocols can generate substantial inter-laboratory variability, affecting organoid morphology, differentiation status, and functional responses. Moreover, the widespread use of animal-derived extracellular matrices such as Matrigel introduces batch-to-batch variability and limits reproducibility, while also representing a significant obstacle for Good Manufacturing Practice (GMP)-compliant production and future clinical applications. Emerging chemically defined extracellular matrices, including PEG-based synthetic hydrogels, recombinant extracellular matrix proteins, and peptide-functionalized xeno-free scaffolds, are being developed as promising alternatives to Matrigel [16]. However, their broader application to intestinal PDOs remains limited by incomplete reproduction of the native extracellular-matrix complexity, variable support of long-term expansion and multilineage differentiation, technically demanding formulation, and the absence of a universally applicable matrix across intestinal regions and disease states. These systems reduce batch-to-batch variability, improve experimental reproducibility, and are more compatible with Good Manufacturing Practice (GMP) requirements. Another important limitation is the incomplete representation of the native intestinal microenvironment. Conventional organoids primarily consist of epithelial cells and therefore lack many of the cellular and structural components that regulate tissue physiology in vivo, including immune cells, fibroblasts, endothelial cells, enteric neurons, vascular networks, and lymphatic drainage. Although recent advances in co-culture systems, organ-on-chip technologies, and bioengineered multicellular models have substantially improved physiological relevance, reproducing the complex bidirectional interactions among epithelial, immune, microbial, stromal, neural, and endocrine compartments remains a considerable challenge. Scalability and clinical implementation also represent significant barriers. The generation and long-term maintenance of patient-derived organoids remain labor-intensive, technically demanding, and relatively expensive. Furthermore, the time required to establish expandable organoid cultures may limit their utility in clinical settings where therapeutic decisions must be made rapidly. The development of automated culture platforms, robotic handling systems, and high-throughput manufacturing pipelines will therefore be essential for integrating organoid-based assays into routine clinical workflows. Additional challenges concern the long-term genetic and phenotypic stability of organoid cultures. Although patient-derived organoids generally preserve the molecular characteristics of the original tissue, prolonged in vitro expansion may introduce clonal selection, genetic drift, or epigenetic changes that could affect experimental reproducibility and the predictive value of drug-response studies. Moreover, long-term expansion may also progressively alter epithelial differentiation status, responsiveness to inflammatory cytokines and microbial metabolites, and clonal composition, emphasizing the importance of limiting passage number and implementing standardized quality-control procedures. Establishing standardized quality-control criteria, including genomic, transcriptomic, and functional validation, will therefore be necessary before organoid-based assays can be broadly implemented in clinical decision-making. Moreover, beyond harmonizing culture conditions, assay standardization should also include the use of application-specific functional readouts. Barrier integrity is commonly evaluated by transepithelial electrical resistance (TEER), FITC-dextran permeability assays, and the expression of tight-junction proteins such as ZO-1, occludin, and claudins. In inflammatory disease models, cytokine profiling (e.g., IL-6, IL-8, TNF-α) and NF-κB activation are widely used to quantify epithelial inflammatory responses. Enteroendocrine studies generally assess hormone secretion, including GLP-1, PYY, and CCK, whereas oncology applications typically rely on cell viability, proliferation, apoptosis, and dose–response analyses to evaluate therapeutic efficacy. In addition, standardized reporting of organoid size, passage number, differentiation status, seeding density, and exposure time would improve reproducibility and facilitate comparisons across laboratories.

Finally, several regulatory and ethical issues remain to be addressed before regenerative organoid therapies can become routine clinical practice. Large-scale GMP-compatible manufacturing, rigorous biosafety assessment, long-term evaluation of engraftment efficiency and tumorigenic potential, together with harmonized regulatory frameworks, will all be required before organoid transplantation or gene-edited organoid therapies can be translated into widespread clinical use. Despite these limitations, the pace of technological innovation remains exceptionally rapid. The convergence of synthetic biomaterials, microfluidic organ-on-chip platforms, single-cell multi-omics, CRISPR-based genome engineering, artificial intelligence, and automated manufacturing is expected to progressively overcome many of the current barriers. These advances will likely transform intestinal organoids from sophisticated experimental models into robust, standardized, and clinically applicable platforms for disease modeling, drug development, regenerative medicine, and personalized healthcare.

## 8. Conclusions

Intestinal organoids have fundamentally transformed the study of gastrointestinal biology by providing human-relevant experimental systems that faithfully reproduce key structural and functional features of the intestinal epithelium. Over the past decade, these models have evolved from simplified epithelial cultures into increasingly sophisticated multicellular platforms capable of investigating host–microbiota interactions, immune regulation, tissue regeneration, and endocrine signaling within a physiologically relevant context. Beyond their value as experimental models, intestinal organoids are progressively reshaping translational gastroenterology by bridging mechanistic research with precision medicine. Their integration with emerging technologies—including single-cell multi-omics, genome editing, artificial intelligence, and organ-on-chip systems—is expected to furtherr enhance their predictive capacity and clinical applicability. Although significant challenges related to standardization, scalability, and regulatory implementation remain, continued technological innovation is likely to accelerate the transition of organoid platforms from research laboratories to routine clinical practice. Overall, intestinal organoids represent one of the most promising experimental systems currently available for understanding gastrointestinal disease and developing individualized therapeutic strategies. Their continued evolution is expected to play a central role in the future of precision gastroenterology and regenerative medicine.

## Figures and Tables

**Table 1:** Clinical applications, strengths, and current limitations of patient-derived organoids in gastrointestinal diseases.

Application	Main Strengths	Current Limitations/Challenges
Disease modeling	Human-derived model that faithfully recapitulates tissue architecture, genetic alterations, and intratumoral heterogeneity	Limited representation of the tumor microenvironment, including vasculature, immune cells, and stromal components
Drug screening	Enables patient-specific functional testing and prediction of treatment response	High cost, variable establishment rates, limited scalability, and lack of standardized drug-testing protocols
Precision medicine	Supports individualized therapeutic decision-making when integrated with molecular profiling	Prospective clinical validation is still limited, and turnaround time may not always be compatible with clinical decision-making
Gene editing and functional studies	Allows investigation of gene function and validation of therapeutic targets using CRISPR-based approaches	Dependence on specialized expertise, technical complexity, and variability among organoid models
Regenerative medicine and tissue engineering	Provides platforms for studying tissue regeneration and epithelial repair	Clinical applications remain largely experimental, with challenges in vascularization and long-term engraftment
Biobanking	Enables long-term preservation of patient-derived samples for translational research and drug discovery	Dependence on Matrigel, lack of standardized biobanking protocols, and quality-control requirements

## Data Availability

No new data were created or analyzed in this study. Data sharing is not applicable to this article.

## Supplementary Materials

The following supporting information can be downloaded at https://www.mdpi.com/article/10.3390/ijms27156949/s1.
