# Supplementary material for: Patient-Derived Organoids in Gastrointestinal Disease: Current Applications, Limitations, and Future Perspectives
Source: Int J Mol Sci. 2026 Aug 2;27(15):6949. doi: 10.3390/ijms27156949 (PMC13466926; doi:10.3390/ijms27156949)
Supplement: Supplementary file 1 [file ijms-27-06949-s001.zip › ijms-4456895-supplementary.pdf]

**Supplementary Table S1.** Comparison of conventional experimental models and patient-derived organoids for gastrointestinal disease research

| Feature | Conventional 2D Cell Lines | Animal Models | Patient-Derived Organoids (PDOs) |
|---|---|---|---|
| **Cellular complexity** | Usually one or few immortalized cell types | Multiple cell types within an intact organism | Multiple epithelial cell types derived from patient tissue |
| **Three-dimensional architecture** | Absent | Present | Present |
| **Patient-specific genetic background** | No | Limited | Preserved |
| **Tumor/epithelial heterogeneity** | Poorly represented | Partial | Maintained |
| **Microenvironment** | Limited | Native tissue environment | Partial; can be enhanced by co-culture or organ-on-chip systems |
| **Immune interactions** | Minimal | Intact | Limited; requires immune co-culture |
| **Microbiota studies** | Limited | Possible | Possible with microinjection, apical-out organoids, monolayers, or gut-on-chip platforms |
| **Drug screening** | High-throughput | Limited by cost and time | Suitable for medium-throughput and personalized drug testing |
| **Genetic manipulation** | Straightforward | More challenging and time-consuming | Efficient using CRISPR/Cas9 and viral approaches |
| **Physiological relevance** | Low | High | High for human epithelial biology |

| Feature | Conventional 2D Cell Lines | Animal Models | Patient-Derived Organoids (PDOs) |
|---|---|---|---|
| **Experimental reproducibility** | High | Moderate | Moderate; influenced by donor variability and culture conditions |
| **Cost and technical complexity** | Low | High | Moderate to high |
| **Major advantages** | Simple, inexpensive, highly scalable | Whole-organism physiology and systemic interactions | Human-specific biology, preservation of disease characteristics, precision medicine applications |
| **Major limitations** | Lack of tissue architecture and cellular diversity | Species-specific differences, ethical concerns, high cost | Lack of vasculature, immune and neural components unless incorporated by co-culture or microfluidic systems |
